# Neuroinflammation and RAMP1: the Role of the Peripheral and Central Nervous System in Tumor Progression

**DOI:** 10.1007/s12013-026-02009-z

**Published:** 2026-02-06

**Authors:** Pedro Daniel Moumesso Cavalcante, Jonas Lucas Dias da Silva, Laryssa Rosy Santos Oliveira, Rikelly Barbosa da Silva, Juliana Acioli Costa Lima, Guilherme de Brito Souza, Davi Ramon da Silva Santos, Júllia Victória Nascimento de Abrantes, Mateus Lima de Farias, Sofia Amancio de Almeida Oliveira, João Paulo Emiliano da Silva, Rafael de Oliveira Calaça Farias, Leonardo Prudêncio Coutinho de Almeida, Salomão Belfort Sparapan de Melo, Sandra Taveiros de Araújo, Raimundo Rodrigues de França-Júnior, José Emerson Xavier, Carlos Alberto de Carvalho Fraga

**Affiliations:** 1 Federal University of Alagoas, Arapiraca Campus, Center for Medical Sciences, Av. Manoel Severino Barbosa, Bom SucessoCEP 57309-005 Arapiraca, AL, Arapiraca, Brazil; 2 CMOL – Computational and Molecular Laboratory, Av. Manoel Severino Barbosa, Bom Sucesso, CEP 57309-005 Arapiraca, AL, Arapiraca, Brazil

**Keywords:** Calcitonin Gene-Related peptide, RAMP, neuroinflammation, Tumor microenvironment, Cell proliferation

## Abstract

Calcitonin gene-related peptide (CGRP) and receptor activity modifier protein 1 (RAMP1) form a critical neuroimmunoendocrine axis in the modulation of neuroinflammation, pain, and tumor progression. CGRP, released by sensory and sympathetic fibers, is a potent vasodilator and nociceptive modulator; its action depends on the receptor composed of CALCRL and RAMP1. In the central nervous system, the activation of microglia and astrocytes and the induction of pathways such as NF-κB and MAPK culminate in the production of proinflammatory cytokines (TNF-α, IL-6), differing from systemic inflammation due to the presence of the blood-brain barrier and the glial microenvironment. Preclinical evidence demonstrates RAMP1 expression in neurons, glial cells, endothelium, and macrophages; CGRP/RAMP1 signaling promotes vascular permeability, cytokine release, and angiogenesis, potentially synergizing with VEGF. In animal models and cell cultures, genetic or pharmacological manipulations of the axis reduce tumor angiogenesis, pro-tumor immune infiltration, tumor growth, and nociceptive behavior. Given the clinical availability of CGRP antagonists and monoclonal antibodies, blocking the CGRP/RAMP1 axis is a promising translational strategy for modulating tumor progression and cancer pain. This review summarizes preclinical evidence on mechanisms and therapeutic feasibility. The article includes preclinical and clinical safety and biomarker studies.

## Basic Concepts about CGRP and RAMP1

Calcitonin Gene-Related Peptide (CGRP) is a 37-amino acid neuroactive peptide belonging to the calcitonin peptide family, which also includes calcitonin itself, amylin (IAPP), and adrenomedullin (ADM), widely recognized for its potent vasodilatory effect [1, 2]. This family shares a distinctive structural feature, an intramolecular disulfide bond between cysteines at positions 2 and 7 at the N-terminal end, forming a six-amino acid ring and crucial for its biological activity [1, 2]. The C-terminal portion of CGRP is amidated, a post-translational modification that increases the peptide’s stability and its affinity for the receptor, protecting it from degradation [1–3].

In humans, CGRP exists in two main isoforms: α-CGRP and β-CGRP. These isoforms are encoded by distinct genes, CALCA and CALCB, located on chromosome 11 [1, 3]. Due to a molecular similarity of approximately 90%, where the difference between the peptide chains is in the sequence of only 3 amino acids between them, these structures exhibit similar vasodilatory biological activity [1, 2]. It is observed that this small variation in structure is related to the site of action of these peptides, with α-CGRP being the main form found in the central and peripheral nervous system, while β-CGRP is found mainly in the enteric nervous system [3].

### CGRP/RAMP1 Axis Signaling

CGRP signaling is mediated by its receptor (CGRP-R), which comprises two subunits: a seven-domain transmembrane protein called Calcitonin-Like Receptor (CLR) and a single-pass transmembrane accessory protein, Receptor Activity Modifier Protein 1 (RAMP1) [1–4]. CLR belongs to the family of class B “secretin-like” G protein-coupled receptors (GPCRs), which also includes receptors for calcitonin, vasoactive intestinal polypeptide (VIP), pituitary adenylate cyclase-activating polypeptide (PACAP), and parathyroid hormone (PTH) [1, 3, 4]. Regarding RAMPs, two more types have been identified in addition to RAMP1: RAMP2 and RAMP3. These molecules exhibit an identity factor of 31% and approximately 56% similarity to each other [4], characteristics that corroborate their functions in regulating the expression of GPCRs, mainly CLR. It was found that the combination of RAMP1 and CLR will originate a CGRP-responsive receptor, while combinations of RAMP2 or RAMP3 and CLR will originate an adrenomedullin receptor [2, 4].

Signal transduction initiated by CGRP is a complex and tightly regulated process that depends on the unique architecture of the CGRP-R. Signaling is not limited to a single intracellular pathway, but encompasses multiple cascades activated after the peptide binds to its canonical receptor complex, composed of CLR and RAMP1 [1–3].

### Receptor Activation and G Protein Binding

The formation of the functional receptor in the plasma membrane is the first step in the process. CLR requires association with RAMP1 for its translocation from the endoplasmic reticulum to the cell surface and for the formation of a high-affinity binding site for CGRP [4]. Cryo-electron microscopy studies have demonstrated the complex topography of this interaction. CGRP binds to a cleft formed by the extracellular domains (ECDs) of CLR and RAMP1, as well as by the transmembrane domains (TMDs) of CLR [5].

The C-terminal end of CGRP interacts primarily with the ECD of the CLR, functioning as an affinity anchor, while the N-terminal ring of the peptide, crucial for activation, inserts itself deeper into the transmembrane bundle of the CLR, inducing the conformational change necessary for G protein coupling [4, 5]. RAMP1 stabilizes the conformation of the CLR and makes allosteric contacts with CGRP, being indispensable for the specificity and potency of the ligand [6, 7]. In many cellular systems, a third protein, the Receptor Component Protein (RCP), is required for efficient coupling of the activated complex to the Gαs subunit of the heterotrimeric G protein [6, 7].

### Canonical and Non-Canonical Intracellular Signaling Pathways

**Fig. 1:**
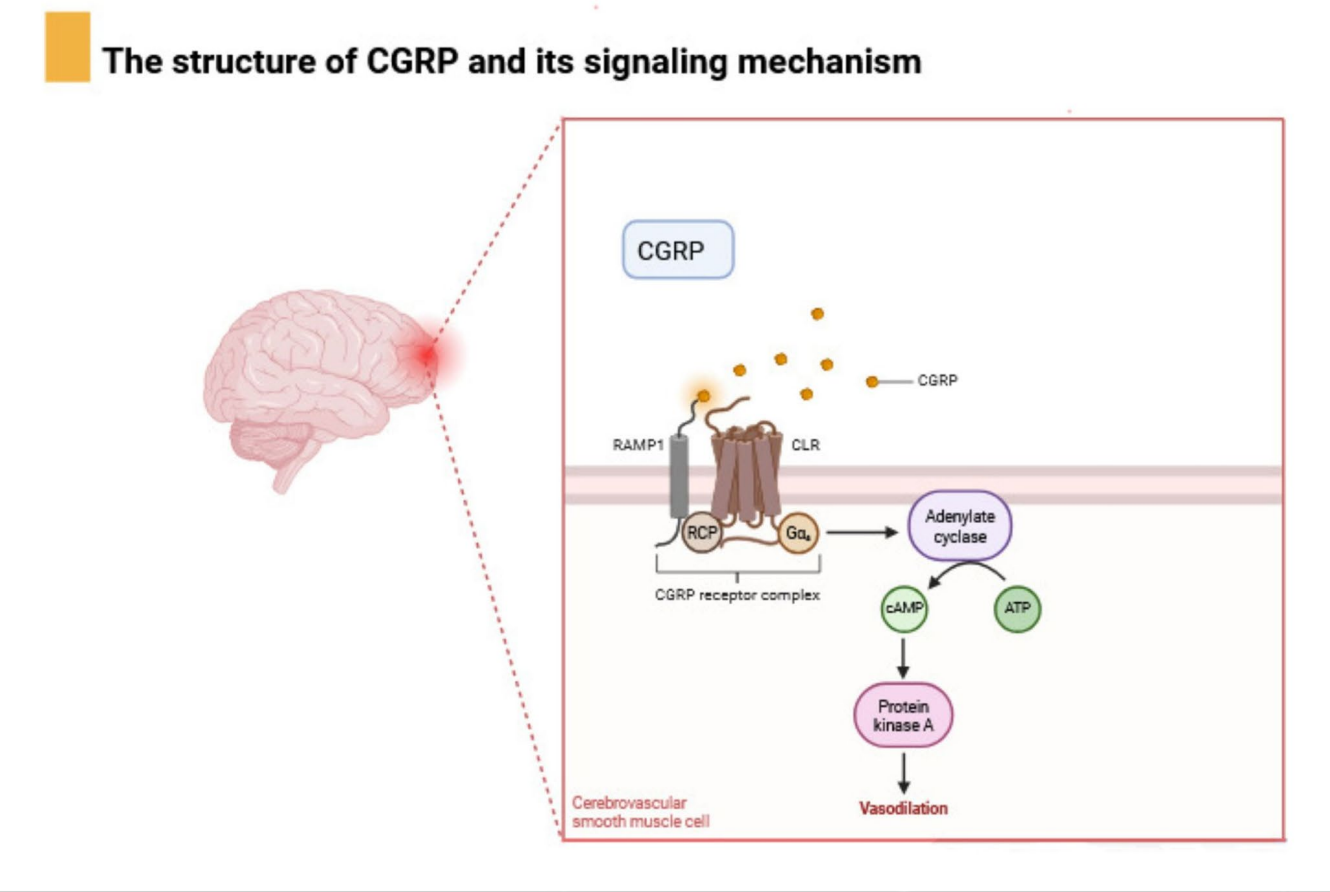
This image illustrates the CGRP receptor complex, comprising CLR, RAMP1, and RCP, situated on a cerebrovascular smooth muscle cell membrane. Upon CGRP binding, the complex activates an intracellular signaling cascade via Gαs​, leading to adenylyl cyclase activation, cAMP production, and subsequent Protein Kinase A activation, ultimately resulting in cellular responses like vasodilation. Created with BioRender

The best characterized and predominant signaling pathway for the CGRP receptor is the canonical pathway mediated by the stimulatory G protein (Gαs). After receptor activation, the Gαs subunit dissociates, binds to, and activates the enzyme adenylate cyclase (AC). AC catalyzes the conversion of ATP to cyclic adenosine monophosphate (cAMP), one of the most abundant second messengers in intracellular pathways [1, 3, 7]. Increased intracellular cAMP levels lead to the activation of Protein Kinase A (PKA), which then phosphorylates a wealth of substrates in sequence. Among these are the cAMP Response Element Transcription Factor, which modulates gene expression, and cytoskeletal proteins associated with ion channels that mediate direct physiological effects, such as vascular smooth muscle relaxation [8] (Fig. 1).

In addition to the canonical Gαs/cAMP/PKA pathway, CGRP signaling can diverge into other cascades, depending on cell type and context. There is evidence that the CGRP receptor can couple to other G protein subunits via three different pathways: Gαi/o, Gαq/11, and MAP kinases.

In the Gai/o pathway, coupling to the inhibitory G protein (Gi/o) can lead to the inhibition of adenylate cyclase, resulting in a decrease in cAMP levels and counterbalancing the Gas pathway [1, 8]. While in the Gaq/11 pathway, in certain cell types, such as osteoblasts, the CGRP receptor can activate the Phospholipase C (PLC) pathway, mediated by Gaq/11. PLC cleaves phosphatidylinositol 4,5-bisphosphate (PIP2) into two second messengers: inositol 1,4,5-triphosphate (IP3) and diacylglycerol (DAG). IP3 induces the release of Ca₂ from the endoplasmic reticulum, while DAG activates Protein Kinase C (PKC), triggering calcium-dependent cellular responses and phosphorylation [1, 4, 8]. Regarding MAP Kinase Pathways, CGRP receptor activation has also been associated with phosphorylation of extracellular signal-regulated kinases (ERK1/2), components of the MAP kinase (MAPK) pathway, which are involved in processes such as cell proliferation, differentiation and survival [1, 8].

### Signal Regulation: Desensitization, Internalization, and Endosomal Signaling

To avoid overstimulation and allow cells to adapt to chronic signals, CGRP receptor activity is finely regulated. After activation, the C-terminal domain of the CLR is phosphorylated by G protein-coupled receptor kinases (GRKs). This phosphorylation promotes the recruitment of β-arrestin proteins [5, 8].

This process has two main consequences. First, it uncouples the receptor from the G protein, a process known as homologous desensitization, which attenuates signaling. Secondly, β-arrestin acts as an adapter for the endocytosis process, promoting the internalization of the receptor-ligand complex from the plasma membrane into the cell via clathrin-coated vesicles [6–8].

After endocytosis, the complex can follow two different paths. It can be directed to lysosomes for degradation, resulting in long-term downregulation of the number of receptors, or it can be recycled back to the plasma membrane, allowing the cell to be resensitized to the stimulus [1, 3, 7, 8]. Recent research has shown that the CGRP receptor continues to signal from the endosomal compartment, activating cAMP cascades that may be spatially and temporally distinct from those originating in the plasma membrane. This endosomal signaling appears to be particularly important for the sustained transmission of pain signals, adding another layer of complexity to the pharmacology of the CGRP/RAMP1 axis [9].

## Role in Neuroimmune and Vascular Signaling

The CGRP/RAMP1 axis constitutes a fundamental apparatus in communication between the nervous, vascular, and immune systems. Its activity is crucial for maintaining tissue homeostasis, as well as being regulatory in pathological processes involving vascular dysregulation and inflammation. The release of CGRP from sensory nerve endings in response to noxious thermal or chemical stimuli triggers a localized and coordinated response known as neurogenic inflammation, a process in which the vascular and immune roles of the peptide are intrinsically interconnected [1, 3].

### Vascular and Hemodynamic Regulation

As previously mentioned, the most recognized function of CGRP is its potent vasodilatory action. CGRP-positive sensory nerve endings form a dense network around arteries and arterioles throughout the body, strategically positioned to regulate vascular tone and local blood flow [10]. The canonical Gαs/cAMP/PKA pathway is crucial for protecting tissues against ischemia and for regulating blood pressure. Pathologically, dysregulation of CGRP/RAMP1 signaling is the starting point of migraine pathophysiology [2, 6, 10]. Excessive release of CGRP in the meningeal vessels during a migraine attack causes painful vasodilation and sensitization of the trigeminal nerve pain pathways. Research shows that blocking signaling of the axis or its receptors is one of the best forms of treatment for migraine [11].

### Modulation of the Immune Response and Neurogenic Inflammation

In addition to its vascular role, CGRP is a modulator of immune function. The response triggered by the neurogenic inflammation process can stimulate the appearance of the cardinal signs of inflammation (redness, heat, edema, pain, and swelling), largely through the modulation of resident and recruited immune cells [1, 10, 11]. The action of CGRP on the immune system depends on the context, and it can exert both pro-inflammatory effects in acute phases and potent anti-inflammatory and immunosuppressive actions in chronic scenarios [1]. In this context, mast cells, dendritic and Langerhans cells, T lymphocytes, and macrophages stand out.

For mast cells, CGRP can induce degranulation, releasing pro-inflammatory mediators such as histamine and serotonin, which contribute to increased vascular permeability and vasodilation [1, 11]. In contrast, CGRP has been shown to have a predominantly inhibitory effect on dendritic cells. In Langerhans cells of the skin, for example, CGRP suppresses their ability to process and present antigens to T lymphocytes, promoting a state of immunological tolerance [12]. Regarding T lymphocytes and macrophages, CGRP signaling can alter T lymphocyte proliferation and differentiation, often shifting the immune response from a pro-inflammatory profile (Th1/Th17) to a more anti-inflammatory profile (Th2). Furthermore, it can modulate macrophage polarization, favoring the M2 phenotype, associated with inflammation resolution and tissue repair [1, 2, 11].

## Neuroinflammation: concept, Mechanisms and Mediators

Neuroinflammation represents a specialized immune response of the nervous system that, in the neoplastic context, transcends its initial protective function, establishing a molecular microenvironment that favors tumorigenesis and malignant progression [13–15]. In glioblastomas (GBM), this dysregulated inflammatory response plays a central role in gliomagenesis, promoting tumor aggressiveness and resistance to treatments [16]. Evidence demonstrates that neuroinflammatory alterations and associated behavioral manifestations, including fatigue and depressive symptomatology, significantly precede evident physical deterioration, suggesting that neural inflammation constitutes a primary event in tumor pathophysiology [17].

The neural tumor microenvironment is characterized by cellular heterogeneity, where the interaction between glial cells and tumor-associated macrophages (TAMs) establishes signaling networks that support neoplastic progression through the coordinated release of cytokines and activation of specific molecular cascades [16].

The compartmentalization imposed by the blood-brain barrier (BBB) fundamentally distinguishes this response from systemic inflammation [15, 18]. During chronic neuroinflammation, BBB disruption allows the infiltration of peripheral immune cells, establishing feedback loops. This process intensifies local inflammation and contributes to an immunosuppressive environment that favors immune evasion and tumor progression [16, 19].

The differences between neuroinflammation in the central nervous system (CNS) and peripheral nervous system (PNS) influence tumor dissemination patterns. While the CNS maintains rigorous mechanisms to preserve post-mitotic neurons through highly controlled inflammatory responses, the PNS exhibits greater permeability and tissue regeneration [15]. This differentiation influences the activation patterns of NF-κB, MAPK, JAK-STAT pathways, and determines the production of inflammatory mediators in the neoplastic microenvironment [15, 18].

Microglia, as the first line of neural defense, exhibit phenotypic plasticity between M1 (pro-inflammatory) and M2 (reparative) states, regulated by pattern recognition receptors (PRRs) that detect pathogen-associated molecular patterns (PAMPs) and damage-associated molecular patterns (DAMPs) [15, 20, 21]. In the tumor niche, microglial morphology directly reflects its functional state: hyperactivation is associated with the retraction of branched processes and the pro-inflammatory phenotype, while the elongation of these processes correlates with anti-inflammatory states [22]. Galectin-3 (GAL-3) stands out as an important mediator of this microglial activation, intensifying inflammatory responses, stimulating the expression of Toll-like receptors (TLRs), and promoting the aggregation of cancerous cells in tumors, establishing a direct connection between neuroinflammation and tumor progression [23, 24].

Modulation of microglial morphology through activation of protein kinase B (Akt) has been identified as a promising strategy for the prevention of neuroinflammation [22]. Compounds that promote the elongation of microglial processes, mediated by the Akt pathway, can suppress microglial activation and associated inflammatory responses that favor cancer propagation [22].

Reactive astrogliosis manifests through A1 (neurotoxic) and A2 (neuroprotective) phenotypes [25, 26]. Astrocytes coordinate local immune responses that, in the tumor microenvironment, facilitate neoplastic progression by releasing pro-inflammatory factors that promote cell survival, resistance to apoptosis, and tissue invasion [14, 16]. Studies indicate that tumor growth is associated with increased mRNA expression of pro-inflammatory cytokines, especially IL-1β and, to a lesser extent, IL-6, in the cortex and hippocampus, directly correlating with observed behavioral changes [17]. Suppression of these cytokines represents a key mechanism in attenuating neuroinflammation and preventing behavioral deficits associated with cancer [22].

Activation of the TLR4 axis triggers signaling via NF-κB, culminating in the production of pro-inflammatory cytokines [24]. Concomitantly, the NLRP3 inflammasome processes IL-1β and IL-18 precursors via caspase-1, establishing a critical axis in neuroinflammatory amplification [27]. The Akt pathway, in turn, plays a fundamental regulatory role, influencing microglial plasticity and modulating inflammatory responses, being a potential target for therapeutic interventions in this context [22]. Pharmacological modulation of this cascade, exemplified by the inhibition of GAL-3 through farnesyl thiosalicylic acid (FTS), demonstrates therapeutic potential by reducing TLR4 expression and neuroinflammation associated with the tumor microenvironment [23].

Interventions targeting neuroinflammation, such as minocycline administration, show a reduction in the expression of inflammatory brain cytokines and a reduction in depressive behaviors, improving functional indicators without a direct impact on tumor volume or muscle mass, highlighting neuroinflammation as an independent therapeutic target in cancer-associated symptomatology [17]. Compounds such as KRIBB11, by promoting the elongation of microglial processes via Akt activation, prevent inflammatory responses and behavioral deficits, reinforcing the relevance of microglial modulation as a therapeutic strategy for neural neoplastic conditions [22].

The production of cytokines and chemokines in the CNS and PNS reflects differences in cell recruitment capacity relevant to tumor evolution. In the CNS, microglia and astrocytes regulate recruitment more restrictively through the BBB³¹, while in the PNS, Schwann cells and resident macrophages facilitate the recruitment of circulating monocytes in a more permissive manner [28, 29]. Cytokines such as IL-1β compromise BBB integrity and modulate neurotransmission, while TNF-α acts through TNFR1 (necroptotic) and TNFR2 (homeostatic) receptors, modulating cell survival [30].

It is proposed that the CCL2 and CXCL10 axes regulate the recruitment of monocytes and T lymphocytes, respectively [31]. This regulation establishes feedbacks that sustain neuroinflammation and maintain conditions favorable to progression and metastasis [31, 32]. Consequently, the elevation of pro-inflammatory brain cytokines is directly correlated with the manifestation of fatigue and depressive behaviors, including anhedonia, preceding significant muscle loss [17].

The receptor formed by the association between CLR and RAMP1 has been the subject of recent studies that highlight its role in modulating neuroinflammation, especially in the context of CGRP signaling [33]. The CLR-RAMP1 complex is essential for the recognition and binding of CGRP, giving CLR the ability to act as a functional receptor for this neuropeptide [33]. Activation of this receptor system promotes mediators that encourage inflammatory cascades capable of significantly influencing the neuroinflammatory response and triggering enhanced biological responses [33] (Table 1).

**Table 1:** Molecular and cellular mediators of neuroinflammation in the tumor niche

Component	Mechanism or associated pathway	Impact on tumor pathophysiology
**Microglia**	Plasticity between the M1 (pro-inflammatory) and M2 (reparative) states.	The tumor niche induces states that favor progression and immune evasion.
**Astrocytes**	Manifestation of reactive phenotypes A1 (neurotoxic) and A2 (neuroprotective).	They release factors that promote cell survival and tissue invasion.
**Cytokines**	Coordinated production of IL-1β, IL-6, and TNF-α.	Elevated blood sugar levels correlate with fatigue and depressive behaviors.
**Galectin-3**	Intensification of the response via Toll-like receptors (TLRs).	It connects microglial activation directly to neoplastic progression.
**Traffic signals**	Activation of the NF-kB, MAPK, JAK-STAT, and Akt cascades.	They regulate the production of mediators and the plasticity of glial cells.

undefinedThis table summarizes the neural immune response mechanisms discussed in the text

CLR-RAMP1 expression is observed peripherally and in arterial smooth muscle cells and potentially in inflammatory cells, but not in central glial cells or neuronal cell bodies of the spinal trigeminal nucleus (STN) [33]. This peripheral location is of critical importance in the tumor context, as it suggests that activation of the CGRP-RAMP1 axis in the neoplastic microenvironment may modulate vascular perfusion, facilitating the supply of nutrients and oxygen essential for tumor growth and proliferation [33].

## RAMP1 as a Modulator of Neuroinflammation

### Evidence of RAMP1 Expression in Neurons and Glial Cells

RAMP expression has long been studied in academia for its ability to alter the conformation of membrane receptors, whose fame necessarily refers to the specificity of the receptor-ligand interaction. The family of these proteins includes three similar ones: RAMP1, RAMP2 and RAMP3 [34]. For receptor adaptation to the CGRP protein, RAMP1 is the associated modulator.

Laboratory analyses were performed to evaluate the presence of RAMP1 protein in the membrane of nervous system cells, both neurons and glial cells, and the results demonstrated reactivity to antigen presentation of the protein in neurons [35–39]. This suggests functional reactivity, indicating a high probability of presence and functionality in the tissue.

Another pharmacological method uses specific antagonists for ligands as a tool to prove the presence of a receptor closely related to the respective ligand, such as the CGRP-RAMP1 relationship. This mechanism allows the tracking of mRNA for the membrane protein, which signals that the cells of that tissue are able to express such a receptor or modulating protein.

Through mRNA screening, it was identified that α-CGRP and β-CGRP structures are expressed in the CNS and PNS and can coexist in a single neuron [40, 41]. CGRP immunoreactivity has been reported throughout the nervous system of α-CGRP^{-/-} mice, suggesting that β-CGRP is present throughout the nervous system.

### Molecular Pathways Activated Via RAMP1 and CGRP

The activation of CGRP function occurs through coupling to the CLR with the receptor-associated modifier protein, that is, it does not have its own specific receptor, but binds to perform its function with a calcitonin receptor adapted for it. Thus, the surface of the target cells of this peptide, such as T lymphocytes, fibroblasts, dendritic cells and mast cells, have CLR-RAMP1 complexes [33].

The basic activation mechanism first involves the coupling of the protein to the membrane receptor, followed by the activation of a signal transduction cascade associated with the Gs protein in the plasma membrane. With the activation of the Gs protein, there is stimulation of AC, leading to an increase in cAMP concentration and this, in turn, increases the phosphorylation of cytosolic targets via PKA, which triggers a metabolic and transcriptional change in the target cell [33].

**Fig. 2:**
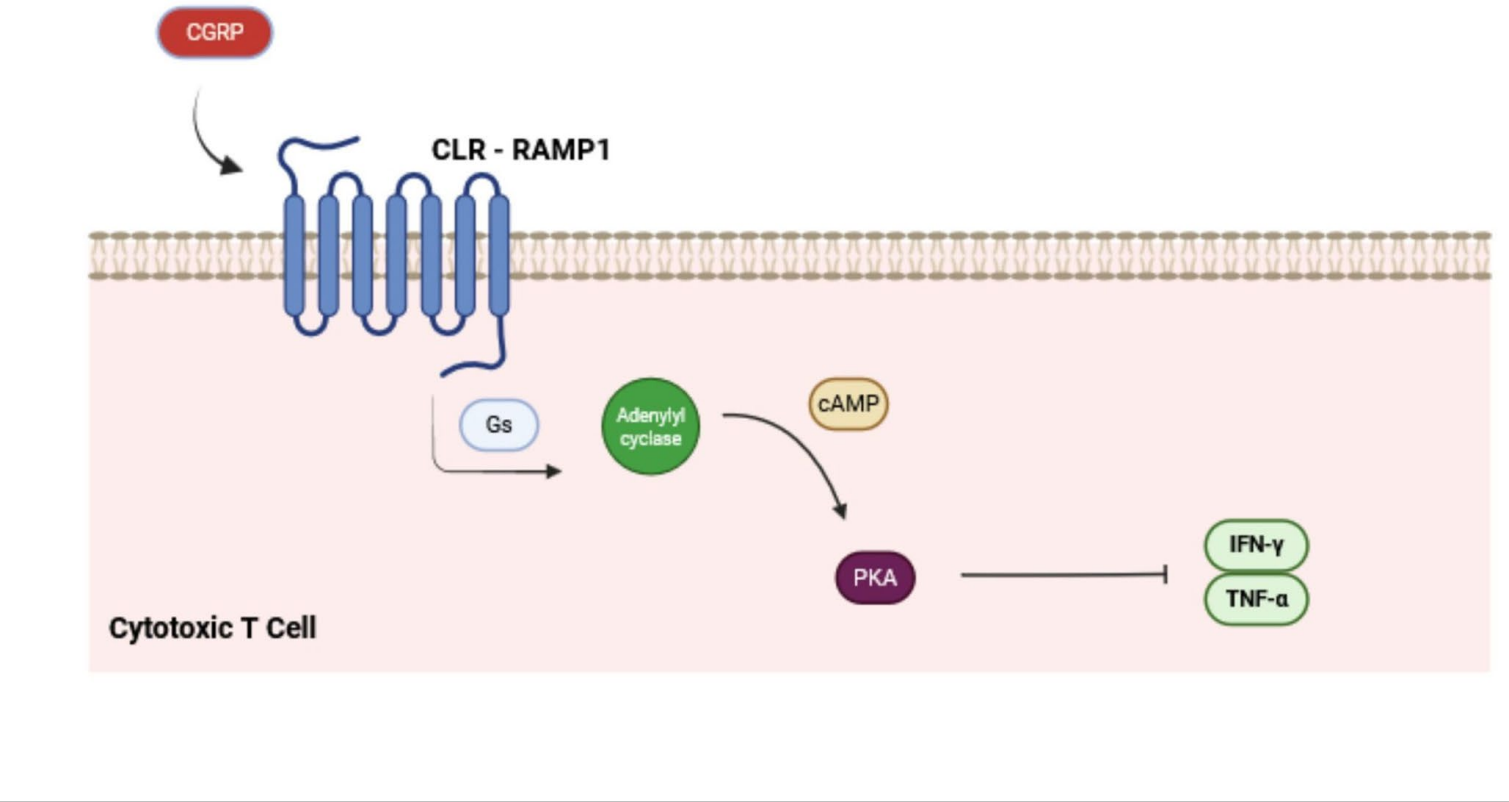
The image above illustrates the signaling pathway of the CLR-RAMP1 complex by CGRP activation in the cytotoxic T cell. The result of transduction cascade is increased transcription of inflammatory cytokines in the cell, such as IFN-y and TNF-a. Created with BioRender

In cytotoxic T lymphocytes, this signal transduction cascade results in altered normal production of inflammatory cytokines, decreasing their apoptotic activity (as shown in Fig. 2). In endothelial cells, CGRP increases vasodilation, contributing to the inflammatory and therefore immune process, although this effect has been shown to be a possible cofactor associated with headache and migraine influenced by hormonal fluctuations in the estrous cycle [34]. In fibroblasts, neuropeptides can modulate their function, with implications for wound healing and are associated with hyperproliferative skin and mesenchymal conditions [33].

### Relationship with the Release of Inflammatory Cytokines (TNF-α, IL-6, etc.)

Considering the activities that the CGRP-RAMP1 axis can influence, the production and release process of inflammatory cytokines also undergoes modifications, altering their proportion in the neuroinflammatory process, which consequently interferes with how immune system cells deal with pathogenic agents such as tumors [40].

A study conducted with laboratory mice examined the expression of CGRP and RAMP1 in individuals with and without application of cells altered for melanoma. The study demonstrated that the tumor promotes axonogenesis, which leads to the development of more nociceptive neurons in the tissue and, therefore, to greater sensitivity to tissue damage. As a result, nerve cells increased the production of CGRP, and an alteration in the responsiveness of CD8 + T cells was observed in individuals with the positive gene for RAMP1 and RAMP1^{-/-}, where those who expressed the receptor modifier showed cellular fatigue in the process of attacking the tumor [40].

The activity of cytotoxic T lymphocytes is important in tumor control because these cells trigger the apoptosis process of cancerous cells, being the main form of neoplasm control and they necessarily act by modulating inflammatory cytokines. Deletion of the RAMP1 gene demonstrated less T cell exhaustion and therefore more efficiency in tumor destruction, while histochemical analysis identified a higher concentration of cellular exhaustion markers (PD-1+, LAG3 + and TIM3+) and therefore reduced capacity to eliminate malignant cells [40].

Along with these results, a reduction in the production of TNF-α and IL-2 was also observed, which directly impacted the tumor necrosis capacity and the proliferative and reactive capacity of CD8 + T lymphocytes, respectively, given the role of these cytokines in the adaptive cellular immune response. CGRP production was reported as a neuronal response to damage, and removing the ability to express RAMP1 proved to be the best alternative to reduce exhaustivity in the context of tumor elimination. It was also seen that CGRP promotes IL-6 biosynthesis induced by IL-1β or TNF-α by a non-transformed fibroblast cell line, through post-transcriptional stabilization of IL-6-specific mRNA [33, 40].

## Interaction between the Peripheral Nervous System (PNS) and Tumors

The peripheral nervous system (PNS) exerts a direct influence on the growth and progression of solid tumors through tumor innervation and the release of neurotransmitters and neuropeptides in the medullary stromal tumor (MST) [41–44]. In preclinical models using rats and mice, it has been demonstrated that tumor growth and progression depend on nerve innervation and that surgical or chemical denervation inhibits tumor advancement [43]. These findings suggest that peripheral neurons infiltrate solid tumors and release neurotransmitters in the MST, activating receptors in cancerous and stromal cells to promote proliferation and dissemination [43]. In humans, it has been observed that in pancreatic cancer the cross-sectional area of intratumoral nerves is significantly larger than in adjacent tissue (*p* = 0.002) and that larger nerves correlate with worse survival (HR ≈ 0.41), indicating a link between innervation and tumor aggressiveness [43].

Recent research in tumor neuroscience shows that multiple types of cancer are capable of recruiting peripheral nerve fibers, a process called tumor innervation, which correlates with more aggressive phenotypes and worse prognosis [44]. Note, however, that the effects vary between tumors: in the pancreas, both sensory and sympathetic nerves promote progression, while parasympathetic cholinergic fibers appear to inhibit the tumor [43]. Many solid tumors have complex innervation, with sensory (nociceptive) and autonomic (sympathetic and parasympathetic) fibers inserting into the TME [35]. These fibers release different chemical ligands that act on receptors of tumor and stromal cells. For example, sympathetic nerves release norepinephrine which activates α/β-adrenergic receptors in cancer cells, increasing, via MAPK/ERK signaling, proliferation, survival and motility [45–48], while parasympathetic nerves may have opposite effects depending on the tumor [43].

Nociceptive neurons that infiltrate tumors can respond to inflammatory cytokines (such as TNFα, IL-1β) and cellular stress by releasing substances such as Substance P and CGRP, similar to the neuroinflammation process described in other contexts [35]. In experiments, CGRP release by sensory neurons increased when tumor cells activated TRPV1 channels (causing immune dysfunction) [35]. Substance P (via NK1R receptor) is known to increase vascular permeability and attract leukocytes in inflammation [41], suggesting that its presence in the TME could facilitate immune infiltration. Furthermore, in vitro studies show that Substance P induces macrophages to assume an M2 “repair” phenotype through the PI3K/Akt pathway [45]. This implies that Substance P release may favor a more immunosuppressive tumor microenvironment. However, this evidence comes primarily from cell cultures, not human tumors, and the exact effects on TME still need confirmation in clinical studies or specific animal models.

The neuropeptide CGRP reduces antitumor immunity; in murine melanoma models, CGRP released by nociceptors binds to the RAMP1 receptor on CD8⁺ lymphocytes, inducing exhaustion and limiting their cytotoxicity [35]. In an orthotopic model of oral carcinoma, denervation or pharmacological blockade of CGRP increased CD8⁺ activation and reduced tumor volume; studies in animals and cell culture corroborate the direct effect of CGRP in decreasing CD8⁺ activation markers [35]. CGRP and substance P also modulate the recruitment and activation of CD4⁺ lymphocytes, interfering with the adaptive immune response; on the other hand, the removal or blockade of sensory fibers increases the infiltration of activated CD4⁺ and CD8⁺ into the tumor, indicating that sensory nerves in the TME promote tumor growth via immunosuppression [47]. CGRP receptor antagonists restore the activity of suppressed conventional T cells and dendritic cells (cDC1/cDC2) in the TME; in animal models, CGRP blockade reduces tumor progression and increases immune infiltration, pointing to the CGRP/RAMP1 pathway as an adjuvant therapeutic target [40].

Sympathetic neurons infiltrate tumors and release NE, which, upon activating α/β receptors in tumor cells, triggers pro-growth cascades (PKA/CREB, ERK), increasing proliferation and motility. In parallel, β2 activation in endothelial cells induces VEGF and angiogenesis, and NE/β-AR signaling in tumor fibroblasts promotes the expression of invasion genes and MMPs, favoring cell remodeling and migration. These mechanisms, together, tend to accelerate tumor growth, a correlation observed clinically in tumors with higher sympathetic density [48]. However, there are context-dependent variations; in pancreatic cancer, for example, cholinergic fibers may inhibit while sympathetic fibers stimulate the tumor, indicating that the effect depends on the intratumoral nerve composition [43].

Furthermore, there is a centralized neuroimmune feedback loop in which afferent sensory pathways inform hypothalamic nuclei, central integration returns signals via cholinergic vagal pathways (modulating cytokines by macrophages via nicotinic α7 receptor), via neuroendocrine outputs (HPA axis and glucocorticoids) and via sympathetic autonomic activation (catecholamines), circadian rhythms and the brain-gut axis also modulate leukocyte recirculation, cDC maturation and macrophage polarization, so that the sum of these signals regulates cytokine/chemokine production, recruitment and efficacy of effector lymphocytes, influencing immune surveillance, chronic inflammation and therapeutic response [46] (Table 2).

**Table 2:** Characterization of the influence of the peripheral nervous system on the tumor microenvironment

Type of innervation	Main binders	Effect on TME
**Nice**	Noradrenaline (NE)	It activates α/β-adrenergic receptors, increasing proliferation and motility.
**Sensory**	CGRP and substance P	It induces CD8 + lymphocyte exhaustion, reducing antitumor cytotoxicity.
**Parasympathetic**	Acetylcholine	Variable effects; in the pancreas, cholinergic fibers may inhibit the tumor.
**TRPV1 + nociceptors**	CGRP	Activation causes a local release of neuropeptides that promote immunosuppression.

The table above summarizes the interactions between infiltrated nerve fibers and the progression of solid tumors

## Role of the Central Nervous System in Tumor Progression

### Brain-Tumor Axis: Hypothalamic-Pituitary Pathways, Stress, and Immunosuppression

The CNS plays a crucial role in regulating multiple biological processes that influence tumor progression, through the integration of neuroendocrine, autonomic, and immune circuits. Communication between the brain and the tumor constitutes a dynamic feedback system, in which neural mediators, such as neurotransmitters, neuropeptides, and hormones, alter the tumor microenvironment and, reciprocally, are modulated by peripheral signals originating from the tumor [49].

The CGRP/RAMP1 axis is central to this neuroimmune interface [50]. Chronic stress activates the HPA axis, whose release of glucocorticoids and catecholamines has immunosuppressive and pro-tumor effects (reduction of CD8⁺ T cells and NK cells) [51]. Sympathetic fibers in the tumor stroma release norepinephrine, activating β-adrenergic receptors that increase VEGF and invasiveness [52]. In parallel, sensory fibers release CGRP, which via RAMP1, promotes a tolerogenic tumor phenotype [53].

The CGRP/RAMP1 axis signaling has been shown to play a relevant role in regulating neuroimmune balance. Activation of this pathway reduces the expression of pro-inflammatory cytokines, such as IL-12 and IFN-γ, and favors the secretion of IL-10 and TGF-β, modulating the activity of macrophages and dendritic cells towards anti-inflammatory profiles [50–53]. In experimental models, CGRP overexpression correlated with CD8⁺ T lymphocyte exhaustion and reduced effector cell infiltration in the tumor microenvironment, indicating a direct contribution of this axis to immune evasion [40].

The hypothalamus plays a role in integrating these signals, coordinating neuroendocrine and autonomic responses that control both systemic homeostasis and tumor behavior [49]. Studies suggest that hypothalamic signaling modulated by CGRP influences the activity of the HPA axis and autonomic nuclei, adjusting the release of hormones and neurotransmitters according to the peripheral inflammatory and metabolic state [46]. This bidirectional communication creates a control circuit in which the CNS acts as a master regulator of the tumor response, affecting proliferation, inflammation, and immunosuppression processes [53–56].

The integration between the CGRP/RAMP1 axis and sympathetic pathways reinforces the systemic nature of neural influence on cancer. CGRP can modulate catecholamine release and simultaneously be released in response to sympathetic stimuli, establishing a feedback mechanism capable of intensifying immunosuppression and tumor growth [57, 58]. This complex neuroendocrine network demonstrates that the brain not only responds to the presence of tumors but actively participates in their progression, adjusting autonomic tone and immune profile according to peripheral stimuli.

### Central Regulation of Peripheral Processes Such as Angiogenesis and Inflammation

The CNS also regulates peripheral processes fundamental to neoplastic progression, such as angiogenesis and chronic inflammation, through the integration between neurovascular and neuroimmune pathways. The modulation of these mechanisms depends on mediators derived from sympathetic and sensory fibers, among which is CGRP, which acts as a molecular link between the nervous system and the tumor microenvironment [59].

In the tumor context, prolonged activation of the CGRP/RAMP1 axis in endothelial cells promotes vascular expansion and stromal remodeling. This signaling induces VEGF expression and activation of the MAPK/ERK and PKA/CREB pathways, resulting in angiogenesis and increased availability of oxygen and nutrients to the tumor [60]. The elevated vascular density, in turn, facilitates metastatic dissemination and reduces the effectiveness of local immune responses [58].

The interaction between the CNS and the tumor vasculature is coordinated by hypothalamic and brainstem centers that control autonomic activity. Through these pathways, the brain adjusts peripheral blood flow and the release of vasoregulatory factors according to hormonal and metabolic stimuli [46]. CGRP, acting as a transmitter of this regulation, plays a dual role, sustaining tissue perfusion in physiological states and, in tumor conditions, perpetuating pathological angiogenesis.

In addition to angiogenesis, the CNS influences peripheral inflammation through neural axes that interconnect stress, metabolism, and immunity. Signaling mediated by CGRP and RAMP1 affects macrophage polarization and dendritic cell maturation, suppressing intense inflammatory responses and limiting lymphocyte activation [61]. This neuropeptide modulation is adjusted by circadian rhythms, controlled by the suprachiasmatic nucleus, which determine diurnal oscillations in the release of neuropeptides and glucocorticoids [46].

The result is a tumor microenvironment in which inflammatory and angiogenic processes are synchronized and controlled by central mechanisms. Prolonged neural stress, with concomitant release of CGRP, catecholamines, and glucocorticoids, amplifies neurovascular communication and contributes to resistance to antitumor therapies [51]. Pharmacological blockade of the CGRP/RAMP1 axis has shown potential in restoring immune function and reducing tumor vascularization, representing a promising avenue for therapeutic intervention [55].

In an integrated manner, the evidence indicates that the CNS controls tumor progression through complex neural and peptide networks, in which the CGRP/RAMP1 axis acts as an essential mediator between central stress, peripheral immunity, and vascular remodeling. This interaction reveals a profound level of coordination between the brain and the tumor, consolidating the concept that oncogenesis is also a systemic neurobiological process [62].

## CGRP/RAMP1 Axis and Tumor Angiogenesis

The synthesis and release of CGRP-related peptide occur predominantly from sensory neurons, whose cell bodies reside in ganglia such as the trigeminal and DRGs [63–65]. The TME is characterized by neurogenesis and the growth of nerve fibers that infiltrate the neoplastic parenchyma and associated vasculature [60]. This innervation provides a critical source of endogenous CGRP directly to the TME [64]. The presence of CGRP in the TME facilitates the vascular supply of the tumor through two interrelated mechanisms: direct vasodilation and active promotion of angiogenesis [64]. CGRP-mediated vasodilation is a direct effect on vascular smooth muscle cells, resulting in increased local blood flow [64]. Although the tumor vasculature is often disorganized and dysfunctional, this increased blood supply is crucial for providing oxygen and nutrients, sustaining the rapid proliferation rate of cancer cells [60].

Functionally, CGRP has demonstrated a robust pro-angiogenic effect, as in in vitro assays, CGRP increased the formation of tubular structures by VECs [64]. Furthermore, the peptide has been shown to promote the proliferation and migration of VECs, processes essential for the formation of new blood vessels [65]. The relevance of these observations was confirmed in vivo, where CGRP was able to increase sponge-induced angiogenesis [64].

In murine models of Lewis lung carcinoma (CLL), CGRP{-/-} mice showed a significant reduction in tumor growth [64]. This reduction in growth was directly correlated with a marked decrease in tumor-associated angiogenesis when compared to wild-type (WT) mice [64]. These data suggest that endogenous CGRP is an indispensable facilitator of tumor angiogenesis [64]. The source of this peptide is unequivocally neural, since the expression of CGRP precursor mRNA was elevated in ganglia (such as the GRD) of tumor-bearing mice [64]. This elevation of expression in ganglia was abolished by surgical denervation, confirming that sensory neurons respond to the presence of the tumor by releasing CGRP to support its supply [64].

Pharmacological modulation of CGRP signaling has also proven effective [40]. Administration of a CGRP antagonist, such as CGRP{8–37}, inhibited the migration and formation of VEC tubes in cultures [65]. In head and neck squamous cell carcinoma (HNSCC) models, blocking CGRP signaling or denervating sensory nerves suppressed tumor growth and angiogenesis [40]. Therefore, CGRP is a key mediator linking sensory innervation to tumor vascular support, representing a promising therapeutic target [65].

Tumor angiogenesis is a complex process driven by the orchestration of multiple growth factors, with VEGF being the most potent and widely studied mediator [65]. Analysis of the interaction between CGRP and VEGF reveals a functional synergy that amplifies the angiogenic response in TME [60]. CGRP not only has direct pro-angiogenic effects, but also regulates the expression of other crucial factors, such as VEGF [64].

The central evidence for synergy lies in the observation that VEGF expression in the tumor stroma is significantly reduced in CGRP(-/-) mice [64]. This finding suggests that the facilitation of tumor growth by CGRP is largely mediated by the upregulation of VEGF expression within the tumor microenvironment [64]. In corneal neovascularization models, blocking CGRP signaling with the antagonist CGRP 8–37 led to a significant reduction in VEGF-A mRNA levels in the tissue [65]. This mechanism may involve activation of the AC/PKA signaling pathway, which has been associated with VEGF induction [64].

This cross-modulation positions CGRP as an integrator of the neurovascular axis, where the peptide released by sensory nerves stimulates stromal or tumor cells to increase VEGF production [60]. This neural-vascular signaling feedback loop creates a highly permissive environment for uncontrolled tumor proliferation [64]. Furthermore, continuous activation of these pathways promotes disorganized vascular expansion, increasing the supply of nutrients to the tumor microenvironment [60]. Consequently, CGRP reinforces both angiogenesis and tumor progression mechanisms, contributing to greater aggressiveness and therapeutic resistance [64].

The synergy of CGRP extends to other pro-angiogenic elements and guidance pathways [60]. The process of neurogenesis and angiogenesis is inherently interdependent in TME, often sharing signaling molecules [60]. Axonal guidance factors, such as Semaphorins and their receptors, Neuropilin-1 (NRP-1), exemplify this interconnection [60]. NRP-1 is notable for being a co-receptor for both Semaphorins and VEGF, and its activation in tumor endothelial cells stimulates angiogenesis [60]. Although CGRP acts on its own specific receptor (CGRP-R), its ability to regulate VEGF expression, which in turn uses NRP-1, illustrates how CGRP fits into the complex web of synergy between neuropeptides and growth factors [60].

Other neurotrophins and neuropeptides, such as NGF and BDNF, also modulate VEGF expression, reinforcing that CGRP is a component of a larger synergistic neural-angiogenic program [60]. Resistance to anti-VEGF therapy, a significant clinical challenge, can be partially explained by the presence of compensatory pathways such as that of CGRP [60]. Therefore, inhibition of CGRP signaling, which acts at the root of VEGF regulation and has intrinsic angiogenic activity, emerges as a promising approach to enhance anti-angiogenic therapies and overcome resistance [40, 64] (Table 3).

**Table 3:** Role of the CGRP/RAMP1 axis in angiogenesis and tumor vascular support

Angiogenic mechanism	Process description	Synergy and molecular regulation
**Vasodilation**	Direct effect on vascular smooth muscle cells.	It increases the supply of oxygen and nutrients to support proliferation.
**Pro-angiogenic activity**	It stimulates the formation of tubes, migration, and proliferation of VECs.	Mediated by sensory innervation that responds to the presence of the tumor.
**VEGF modulation**	Upregulation of VEGF expression in the tumor stroma.	AC/PKA signaling is associated with the induction of this growth factor.
**Neurovascular integration**	Interaction with axonal guidance factors such as NRP-1.	It creates a permissive environment for vascular expansion and metastatic spread.

The table above summarizes the mechanisms by which CGRP signaling facilitates neoplastic blood supply

## Implications of the CGRP/RAMP1 Axis in Tumor Pain and Neuroimmune Modulation

### Neuropathic Pain Associated with Cancer and RAMP1 Involvement

Pain is one of the clinical manifestations and a major limiting factor in cancer patients, profoundly affecting quality of life and functionality [66]. Previously treated as a secondary symptom, primary cancer pain, more precisely that which manifests before treatment, has been progressively re-evaluated as a prognostic marker of adverse outcomes [66]. In aggressive tumors, such as oral squamous cell carcinomas and pancreatic adenocarcinomas, pain before treatment is associated with advanced locoregional disease and, consistently, with perineural invasion (PNI) [66]. This correlation suggests that pain does not only represent an indirect consequence of the effect of tumor mass, but may reflect the clinical manifestation of an active neurobiological process closely linked to disease progression [66, 67].

This phenomenon is central to the expanding field of “Cancer Neuroscience,” which studies the complex network of crosstalk between the nervous system and tumor cells [55, 67]. Recent evidence demonstrates that this is an intense exchange interaction between both systems. On the one hand, tumor cells actively secrete neurotrophic factors, such as NGF and BDNF [66, 67]. These factors function as chemotactic signals that induce axonogenesis, sprouting, and infiltration of new nerve fibers (sensory, sympathetic, and parasympathetic) into the TME [67]. In turn, these infiltrated nerve fibers become active participants in tumorigenesis, releasing neurotransmitters such as glutamate, acetylcholine, and norepinephrine, which directly promote tumor proliferation, angiogenesis, and immune evasion [67].

In this set of neural mechanisms, the sensory nervous system, responsible for nociception (pain perception), plays a major role [66]. Nociceptors in the TME are activated by a range of tumor mediators, including NGFs themselves, inflammatory mediators and EVs [66]. This neuronal activation triggers a dual and simultaneous signaling mechanism. The afferent (orthodromic) propagation of the action potential travels to the central nervous system, being interpreted by the brain as the sensation of pain [66]. Despite this, the same activation causes an efferent (antidromic) propagation, resulting in the local release of neuropeptides by sensory nerve endings, specifically from neurons expressing TRPV1, directly in the TME [66].

The importance of the RAMP family in oncology was recently highlighted by a pan-cancer analysis, which identified RAMPs (including RAMP1 and RAMP3) as prognostic biomarkers and molecular hubs widely involved in immunological processes in multiple tumor types, such as glioma [68].

The CGRP-RAMP1 axis is not a pathological mechanism in itself, but rather the co-optation of a physiological neuroimmune pathway [69]. In healthy barrier tissues, such as skin and mucous membranes, the CGRP-RAMP1 axis is used by the somatosensory nervous system to regulate adaptive immunity to commensal microbiota. Neurons release CGRP which binds to RAMP1 expressed on CD8 + T lymphocytes, modulating their activation state and restricting inflammatory responses (such as those mediated by Type 17 interleukins) to maintain homeostasis [69]. Malignant neoplasia, by inducing sensory axonogenesis, hijacks this immunological brake pathway.

In TME, CGRP released by neural cells binds to RAMP1 expressed on TILs (69). This neuroimmune signaling suppresses the effector activity of T cells and promotes an exhaustion phenotype, allowing the tumor to evade destruction by the immune system [69, 70]. Therefore, the same biological event, the activation of nociceptors, jointly generates the clinical symptom of pain and the biological mechanism of immunosuppression. This coupling establishes a vicious cycle: the presence of the tumor causes pain through neural activation, and this same neural activation protects the tumor by suppressing immunity. This interdependence, where the tumor becomes dependent on neural signals for its survival and dynamic remodeling, has been conceptualized as a form of CGRP-mediated “neural addiction” [70–72].

### Possible Feedback Effects on Tumor Trogression

The coupling between nociception and immune modulation can establish a positive feedback loop relevant to tumor progression [66]. This circuit should not be interpreted as a static event, but as a dynamic and potentially self-amplifying process, in which the tumor and the nervous system mutually co-opt each other. This interdependence has been described as a form of “neural dependence” mediated by CGRP [70, 73], in which the tumor not only benefits from neural signaling, but becomes physiologically dependent on it for its continuous remodeling and survival [70, 74].

Evidence suggests that this feedback loop can be actively initiated by the tumor cell [67]. To favor the supply of neuropeptides on which it becomes dependent, such as CGRP, the tumor secretes a set of neurotrophic elements, including NGF and BDNF [66, 67]. These factors function as potent chemotactic signals capable of inducing axogenesis, sprouting and infiltration of new nerve fibers, predominantly CGRP nociceptors, directly into the TME [66, 67]. In this way, the nerve becomes integrated into the TME as a functional structural component [65].

Once recruited, these nerve fibers can be activated by tumor mediators, releasing their CGRP [66, 73]. As established, this CGRP binds to RAMP1 expressed in TILs, functioning as an immune brake that moderates its activation and restricts the antitumor response [69]. This local immunosuppression, mediated by the CGRP-RAMP1 axis, is the primary benefit that the tumor derives from this “neural dependence“ [70, 74]. However, the pro-tumor effect is not limited to CGRP; these co-opted nerve fibers also release other classic neurotransmitters, such as glutamate and norepinephrine, which directly promote proliferation, angiogenesis, and immune escape [67].

The feedback loop can intensify as the tumor, protected from immune surveillance, begins to secrete increasing amounts of NGF and BDNF [66, 67]. This increased secretion favors the recruitment of sensory nerve fibers, which, in turn, leads to an even greater release of CGRP. This increase in CGRP concentration solidifies the immunosuppressive TME, suggesting greater tumor progression [68, 69]. Pain itself, mediated by the afferent pathway [66], can be interpreted as a clinical indicator that this efferent pro-tumor feedback loop is active and accelerating, indelibly linking the patient’s symptom to the mechanism of progression of their disease [66, 70] (Table 4).

**Table 4:** Summary of the CGRP/RAMP1 axis in pain and tumor progression

Component/Mechanism	Description of the Interaction in TME	Clinical/Biological Implication
**Perineural Invasion (PNI)**	Correlation between pre-treatment pain and tumor infiltration in nerves (e.g., oral squamous cell carcinoma, pancreas).	A marker of adverse prognosis and advanced disease.
**Neuro-Tumoral Crosstalk**	Tumors secrete **NGF/BDNF** (axonogenesis); nerves release **CGRP**, glutamate, and norepinephrine.	Establishment of “Neural Dependency” ( *Neural Addiction* ).
**Antidromic Signaling**	Activation of TRPV1 + nociceptors leads to the local release of CGRP by sensory nerve endings in the TME.	Direct coupling between the sensation of pain and local immunosuppression.
**Immune Modulation (RAMP1)**	CGRP binds to RAMP1 on **TILs** (CD8 + T lymphocytes).	Suppression of effector activity and induction of a cellular exhaustion phenotype.
**Feedback Loop**	The tumor recruits nerves to obtain CGRP, which in turn facilitates immune evasion and further growth.	A self-amplifying process that accelerates disease progression.

The table above summarizes the main preclinical studies that validate the CGRP/RAMP1 axis as a therapeutic target

## Experimental Studies: Animal Models and Cell Cultures

Experimental studies using CGRP^{-/-} mice and genetic manipulations that inhibit RAMP1 expression have provided consistent evidence that the CGRP-RAMP1 axis can influence the tumor microenvironment, modulating the inflammatory response and metastatic progression [66, 75, 76]. Among these studies, in murine models of lung cancer, disruption of the CGRP gene reduced angiogenesis and promoted tumor regression [1, 66]. Similarly, RAMP1 expression is specifically elevated in human prostate cancer [74], and its silencing decreased cell proliferation and tumorigenicity in vitro and in vivo [75]. Therefore, these findings reinforce the therapeutic potential of the pathway and suggest that drugs already developed to block it can be repurposed for oncological use.

Therefore, in the study by Toda et al. (2008) it was observed that both the development of the neuronal system of tumor angiogenesis and tumor growth are facilitated by CGRP [1, 66, 77]. These authors demonstrated, using murine experimental models of lung cancer, that the interruption of the CGRP gene, associated with the implantation of Lewis lung carcinoma cells, resulted in a reduction in angiogenesis and tumor growth due to the negative regulation of VEGF expression, a protein crucial for the formation of new blood vessels, confirming the pro-tumor role of the peptide [1, 66, 77].

Furthermore, in studies conducted by Toda et al. (2008), the authors also observed that the use of CGRP antagonists or denervation of the sciatic nerves (L1-5) reduced the levels of the peptide and, consequently, suppressed the growth of Lewis carcinoma [66]. These findings were compared to those of wild-type (WT) mice with CLL, without genetic manipulation, which had amplified levels of CGRP precursor mRNA in the dorsal root ganglion and showed increased angiogenesis and greater tumor growth, corroborating the hypothesis that CGRP participates in the vascular supply of the neoplasm [72]. Thus, the reduced angiogenesis observed in CGRP- ^/^ - animals suggests that the presence of this endogenous peptide plays a significant role in neoplastic progression [1, 66] .

Furthermore, Toda et al. (2008) suggest that CGRP relevant to tumor progression may be derived from neuronal systems, including primary sensory neurons [66]. This hypothesis was initially tested in vitro, when human umbilical vein endothelial cells (HUVECs) were co-cultured with fibroblasts and it was observed that CGRP promoted the formation of new vessels. Subsequently, the pro-angiogenic activity of the peptide was tested in vivo, in CGRP^{-/-} mice implanted with Lewis lung carcinoma (LLC) cells in dorsal subcutaneous tissues, resulting in significantly reduced tumor growth. Thus, the authors suggest that CGRP may become a potential therapeutic target for the treatment of cancers, given its important role in stimulating tumor angiogenesis [66].

Complementing the findings of Toda et al. (2008), other studies have also investigated the relevance of the CGRP-RAMP1 axis in tumor progression. In particular, Logan et al. (2013) demonstrated that RAMP1 expression is specifically elevated in human prostate cancer [75], supported by experiments in NKX3.1^{-/-} mice, a tumor suppressor gene associated with prostate development [77–80]. Under these conditions, the loss of the gene’s repressor function results in amplification of RAMP1 activity and consequent activation of the CGRP-RAMP1 axis [4, 79, 80], which stimulates angiogenesis, cell proliferation and immune evasion, favoring tumorigenesis. These results reinforce that the activation of this axis acts as a facilitating mechanism for tumor progression and maintenance of the malignant phenotype.

Thus, like RAMP1, CGRP also modulates the tumor microenvironment, influencing angiogenesis and the inflammatory response. In other experiments, Mcllvried et al. (2022) investigated its effects on oral squamous cell carcinoma, a cancer highly innervated by sensory neurons that release CGRP [76, 81, 82]. The analysis was performed using a murine model of tongue tumor transplantation and CALCA^{-/-} mice, the gene that encodes CGRP, observing that there was a reduction in tumor growth and an increase in cytotoxic CD4 + and CD8 + T cells compared to wild-type animals, in which CD4 + T cells exhibited higher RAMP1 expression [83]. These findings indicate that the immune system may favor tumor progression by activating the CGRP-RAMP1 axis [84] and reinforce its therapeutic potential, suggesting the possible readaptation of drugs already used against migraine for oncological applications (Table 5).

Therefore, the integration of experimental findings from the analyzed studies suggests that the CGRP-RAMP1 axis plays a multifaceted role in tumor progression, encompassing neuroimmune signals and modulating essential processes such as angiogenesis and weakening antitumor immunosurveillance. Furthermore, this axis is also a potential therapeutic target in highly innervated neoplasms, such as oral squamous cell carcinoma, which could benefit from the repurposing of drugs used in the treatment of neurological disorders, such as CGRP antagonists used in migraine. However, clinical studies evaluating other types of tumors, as well as the systemic effects of blocking this axis in humans, are necessary to determine if such manipulations are, in fact, safe and effective in oncological practice.

**Table 5:** Synthesis of experimental evidence and knockout model results

Study (reference)	Experimental model	Main results observed
**Toda et al. (2008)**	LLC in CGRP-/- mice.	Significant reduction in angiogenesis and tumor growth.
**Logan et al. (2013)**	Prostate cancer and loss of N, X3.1.	Elevated RAMP1 levels promote tumorigenicity and cell proliferation.
**McIlvried et al. (2022)**	OSCC in CALCA-/- mice.	Increased infiltration of activated CD4 + and CD8 + cells into the tumor.
**Studies with antagonists**	Use of CGRP 8–37 and gepants.	Inhibition of cell migration and restoration of immune function in the TME.

The table above summarizes the main preclinical studies that validate the CGRP/RAMP1 axis as a therapeutic target.

## Therapeutic and Pharmacological Perspectives

Calcitonin gene-related peptide antagonists exert their antagonistic action by binding to the CGRP receptor. These antagonists were the first oral drugs developed for the prevention and acute treatment of migraine and can be divided into monoclonal antibodies and small non-peptidic molecules, also known as gepants [82]. The first generation, represented by olcegepant and telcagepant, raised concerns regarding liver toxicity and low oral bioavailability, with effect in migraine prevention [82]. The second generation, represented by rimegepant, ubrogepant and atogepant, shows efficacy in the acute treatment of migraine. The third generation, represented by Zavegelpant, is characterized by having several routes of administration, such as intranasal, for example [82].

**Fig. 3:**
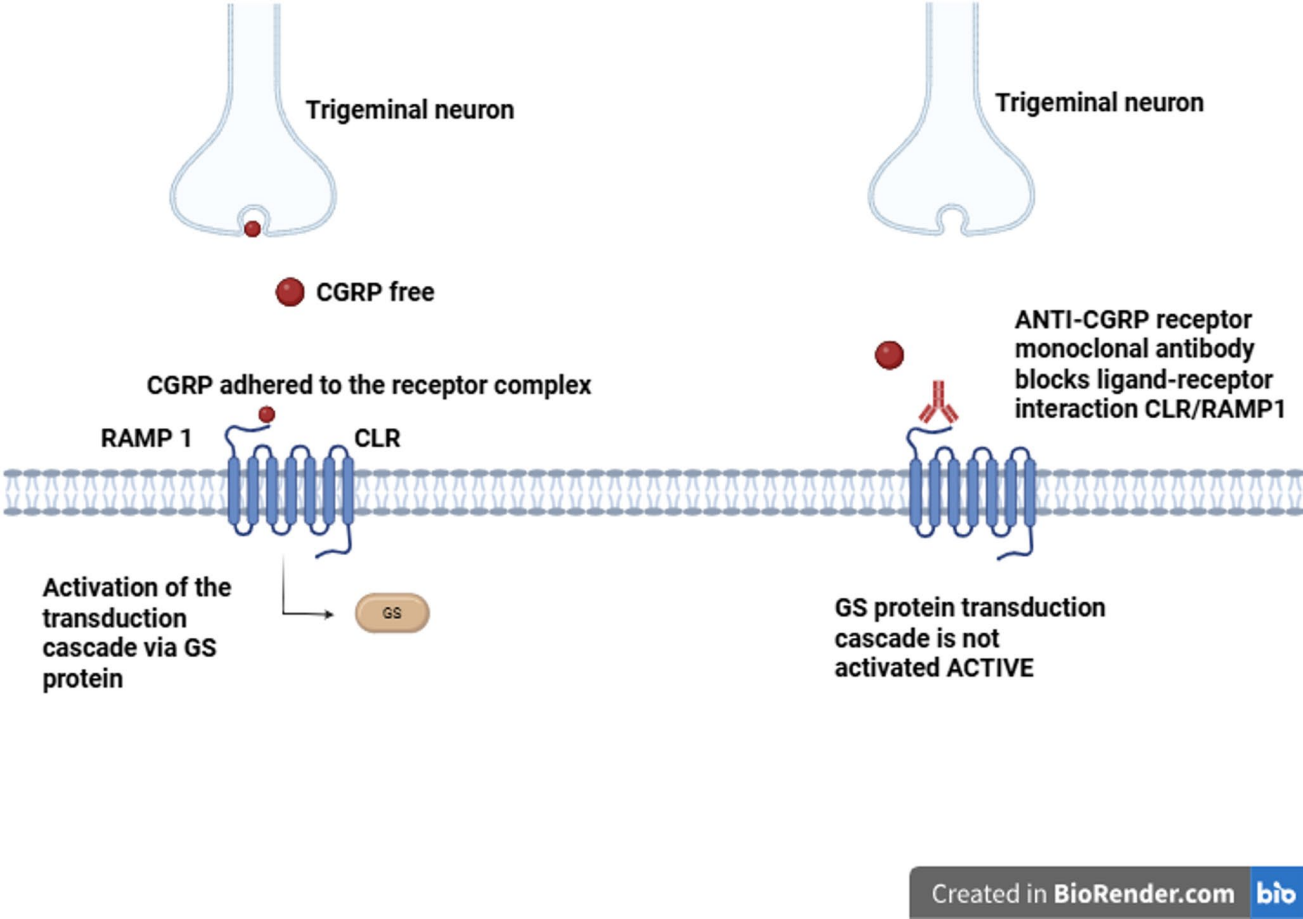
The figure illustrates the mechanism of action of CGRP and the effect of pharmacological blockade of its receptor by monoclonal antibody in the peripheral trigeminovascular system. On the left, the release of CGRP by the peripheral trigeminal neuron is shown, followed by its binding to the RAMP1 receptor and subsequent activation of the GS protein-dependent transduction cascade, which results in increased nociception. On the right, the blockade of this pathway is demonstrated by the binding of the anti-CGRP receptor monoclonal antibody to the CLR/RAMP1 complex, resulting in the non-activation of the Gs protein-mediated transduction cascade. Created with BioRender

Gepants are non-peptide antagonists of the CGRP receptor, blocking the CGRP-receptor interaction in which the transduction pathway mediated by Gs protein, adenylate cyclase and cAMP is impeded, thus interrupting the vasodilatory and pain sensitizing effects that CGRP normally causes in nerve and vascular tissues, more specifically in the context of migraine [83] (Figure 3).

Gepants require optimal bioavailability to be effective: rimegepant requires a clinical dose of 75 mg orally, ubrogepant 100 mg orally, atogepant 60 mg orally, and zavegepant 10 mg intranasally, with plasma concentrations of 0.10 µM, 0.17 µM, 0.13 µM, and 0.05 µM respectively [83]. That is, these concentration levels represent an occupancy of 70 to 90% of CGRP receptors (CLR/RAMP1) [83]. At these concentrations, almost complete inhibition of CGRP-induced vasodilatory responses is observed [83]. Thus, the therapeutic window of efficacy of gepants is around 50–200 nanomolecules (nM) [83].

Compared to gepants, monoclonal antibodies (mAbs) are large proteins, heterodimeric antibodies, designed to block target molecules [84]. These antibodies also act as CGRP antagonists, but unlike gepants, which block the interaction of CGRP with its receptor by competing for binding sites (rapid, reversible and short-lived action), antibodies, in turn, act outside the cell by sequestering the CGRP ligand or blocking the receptor in an irreversible or prolonged manner, with a half-life of weeks and a sustained effect [84].

Four monoclonal antibodies were developed; erenumab acts on the CGRP receptor, while fremanezumab, galcanezumab, and eptinezumab act on the CGRP ligand itself. These medications have proven effective in preventing migraines [84]. Erenumab binds to the CLR-RAMP1 domain interface to block it and prevents the action of other peptides such as adrenomodulin and intermedin, which can act on the CGRP receptor, thus being characterized as an antireceptor [84].

Anti-ligand mAbs (fremanezumab, galcanezumab, and eptinezumab) bind directly to the same region of the CGRP ligand that binds to the receptor, resulting in a specific ligand blockade [84]. The main hypothesis for the site of action and preventive effect of anti-CGRP mAbs in general is that, due to their large size, they act peripherally in locations such as the dura mater, dural blood vessels, and trigeminal ganglion [84]. In these locations, they exert their pain inhibition mechanism; for example, in rat models, fremanezumab selectively inhibited the activation of Aδ-type afferent meningeal nociceptors [84].

There is fundamental preclinical evidence pointing to a relationship between pain reduction in cancer cases and the use of CGRP antagonists, since pain perception is related to the mechanism of this polypeptide. Taking oral cancer as an example, due to its higher prevalence and severity of pain, it is observed that pain is generated in the periphery, within the cancerous microenvironment [85]. Primary afferent (peptidergic) neurons express CGRP, which in turn is well established as a mediator of trigeminal pain (migraine) and neurological inflammation [85].

CGRP is closely related to pain perception in oral cancer, since oral cancer cells express components of the CGRP receptor (RAMP1 and CALCRL). This expression is associated with poorer overall survival and perineural invasion and lymph node metastasis. In mice, tumor innervation by neurons and neurites immunoreactive to CGRP (neurons that produce or react to CGRP) has been observed [85]. Cancer increases anterograde transport (movement of substances within the neuron from the cell body to the ends of the neurites) in the axons that innervate the tumor, which supports neurogenic secretion as a source of CGRP in the oral cancer microenvironment [85]. This activation increases pain perception through the sensitivity of nociceptors [85].

CGRP antagonism reverses oral cancer nociception in preclinical pain models [85]. The action of CGRP antagonists (both gepants and monoclonal antibodies) has been studied in combating this pain mechanism in cancer patients [85]. In preclinical tests with laboratory rats, olcegepant reduced mechanical allodynia (painful sensation with stimuli that normally does not cause pain, such as touch) of cancer for at least 6 h [85].

Given this, there is clear potential focused on repurposing CGRP antagonists from migraine treatment to cancer pain management (such as oral CGRP), due to the fundamental role CGRP plays in tumor nociception. Repurposing these drugs would be of great value in improving the quality of life of cancer patients, thus expanding the range of medications capable of generating positive effects in oncological conditions.

Drug repositioning seeks to find new applications for existing drugs [86]. For repositioning to occur, there are some steps to be carried out such as hypothesis generation, followed by efficacy determination and subsequent clinical trials [86]. Despite the high potential of CGRP antagonists in the management of cancer pain, some obstacles hinder the effective implementation of their clinical translocation.

This potential has been hampered primarily by financial factors, which are one of the main obstacles. Repositioning has received less investment compared to the development of new chemical formulations, mainly because it offers limited financial returns to the pharmaceutical industry [86]. Thus, limiting prospects for new treatments.

Bioavailability, as well as tissue distribution of these drugs in the tumor microenvironment, are also considered as obstacles to pharmacological translation. Despite the proven action of these drugs in neurological conditions, there is still insufficient elucidation regarding the plasma quantities for interaction at adequate therapeutic levels in human peripheral tumor tissues, and such difficulties are widely described in pharmacological repositioning strategies in oncology [86].

Furthermore, the translational gap between preclinical models and clinical applications is a limiting factor. Many positive results in in vitro studies and in animal models do not always materialize in humans, due to physiological and pharmacokinetic differences and the complexity of tumor microenvironments, which compromises efficacy and hinders investment by the pharmaceutical industry [86].

## Conclusions and Future Perspectives

In conclusion, the CGRP/RAMP1 axis plays a broad role in communication between the nervous system, the immune system, the vasculature, and the tumor microenvironment, participating in both neuroinflammation and cancer progression. CGRP, known for its strong vasodilatory effect and its modulating action on the neuroimmune response, is released by nociceptive fibers that infiltrate solid tumors. This release contributes to multiple pro-tumor effects, including TCD8^+^, local immunosuppression, increased inflammatory cytokines (TNF-α, IL-6, and IL-2), and stimulation of angiogenesis through VEGF induction. The strong expression of RAMP1 in neurons reinforces the notion that tumor pain is not just a symptom, but an active component of tumor dynamics.

In addition, animal models support the functional impact of this axis, showing that blocking CGRP or removing RAMP1 reduces tumor growth, decreases angiogenesis, and restores immune surveillance, even enhancing anti-VEGF therapies. Despite recent advances, important gaps remain in our understanding of CGRP/RAMP1 dynamics, especially regarding its role in the central nervous system, its presence in glial cells, and tumor heterogeneity.

Thus, the CGRP/RAMP1 axis is configured not only as a biomarker of neuroimmune interaction in the context of neoplasms also behaves as a strategic therapeutic target. Antagonists already approved in other clinical conditions represent a promising opportunity for therapeutic repositioning, with the potential to restore immunosurveillance, reduce angiogenesis, and improve tumor control. However, translating these findings into oncological practice requires robust clinical studies that evaluate safety, efficacy, and possible systemic effects, in order to transform the blockade of this axis into an effective tool to improve the prognosis and quality of life of cancer patients (Table 6).

**Table 6:** Pharmacological perspectives and repositioning strategies

Drug class	Examples of agents	Proposed mechanism of action	Clinical potential/obstacles
**Gepants**	Rimegepant, ubrogepant, atogepant.	Non-peptidic competitive antagonists of the CLR/RAMP1 receptor.	Rapid receptor occupancy; potential for managing cancer pain.
**mABS (Antireceptor)**	Erenumab.	It binds to the CLR-RAMP1 interface, blocking multipeptide signaling.	Sustained and prolonged effect due to long plasma half-life.
**mABS (Anti-bonding)**	Fremanezumab, galcanezumab.	They sequester the circulating CGRP ligand, preventing it from binding to the receptor.	Block the specific ligand by acting on peripheral sites such as the dura mater.
**Translational obstacles**	–	–	Financial limitations and gaps between animal and human models.

The table above compares classes of drugs targeting the CGRP/RAMP1 axis

## Data Availability

No datasets were generated or analysed during the current study.

## Abbreviations

Abbreviations: Meaning
AC: Adenylate cyclase
ADM: Adrenomedulin
AKT: Protein kinase B
BBB: Blood-brain barrier
cAMP: Cyclic AMP
CCL2: Chemokine ligand 2 with CC motif
CGRP: Calcitonin gene-related peptide
CGRP-R: CGRP receptor
CLR: Calcitonin-Like Receptor
CNS: Central nervous system
CXCL10: Chemokine ligand 10 with CXC motif
DAMP: Damage-associated molecular pattern
DRG: Dorsal root ganglia
ECD: Extracellular domain
FTS: Farnesyl thiosalicylic acid
GAL-3: Galectin-3
GBM: Glioblastoma
GRK: G protein-coupled receptor
HPA: Hypothalamus-pituitary-adrenal axis
IAPP: Amylin
IL: Interleukin
JAK-STAT: Janus Kinase/Signal Transducer and Transcription Activato
MAPK: Mitogen-activated protein kinase
mRNA: Messenger RNA
NE: Noradrenaline
NF-κB: Nuclear Factor Kappa B
NLRP3: Nucleotide-Binding Domain, Leucine-Rich Family, Pyrin-Containing Domain 3
PAMP: Pathogen-associated molecular pattern
PKA: Protein Kinase A
PNS: Peripheral nervous system
PRR: Pattern Recognition Receiver
PTH: Parathyroid hormone
RAMP: Receptor-associated modulating protein
RCP: Protein component of the receptor
TLR: Toll-like receptor
TLR4: Toll-like receptor 4
TAMs: Tumor-associated macrophages
TMD: Transmembrane domain
TME: Tumor microenvironment
TNF: Tumor necrosis factor
TNFR: TNF receptor
VEC: Vascular endothelial cells
VEGF: Vascular Endothelial Growth Factor
VIP: Vasoactive intestinal polypeptide
